# Statin use and hip fractures in U.S. kidney transplant recipients

**DOI:** 10.1186/s12882-017-0559-9

**Published:** 2017-05-01

**Authors:** Chandan Vangala, Colin R. Lenihan, Maria E. Montez-Rath, Sumi Sukumaran Nair, Sankar D. Navaneethan, Venkat Ramanathan, Wolfgang C. Winkelmayer

**Affiliations:** 1 Baylor College of Medicine, Section of Nephrology, One Baylor Plaza, BCM 620 – 11D32.5, Houston, TX 77030 USA; 2 Division of Nephrology, Stanford University School of Medicine, 777 Welch Road Suite DE, Palo Alto, CA 94304 USA; 3 Division of Nephrology, Department of Medicine, Stanford University School of Medicine, 1070 Arastradero Road # 3C11C, Palo Alto, CA 94304 USA; 4 Mayo Clinic Arizona, 5777E Mayo Blvd, Phoenix, AZ 85012 USA; 5 Baylor College of Medicine, Section of Nephrology, One Baylor Plaza, Ste 100-37D, Houston, TX 77030 USA; 6 Section of Nephrology, Michael E. DeBakey VA Medical Center, 2002 Holcombe Boulevard, #111-J, Houston, TX 77030 USA; 7 Baylor College of Medicine, Section of Nephrology, One Baylor Plaza, BCM 395, Houston, TX 77030 USA

**Keywords:** End-stage renal disease, Hip fracture, Outcomes, Drug safety, Case-control, USRDS

## Abstract

**Background:**

Basic and translational research supports beneficial effects of statins on bone metabolism. Clinical studies suggest that statin use may reduce the risk of hip fractures in the general population. Whether statin use is associated with hip fracture risk in kidney transplant recipients, a particularly high-risk group for this outcome, is unknown.

**Methods:**

From the U.S. Renal Data System (2007–2011), we identified all hip fracture events recorded in Medicare billing claims of first-time kidney transplant recipients. We then matched all cases to an unlimited number of controls on age (±3 years), sex, race (black vs. non-black), and time since transplant. Cases and controls were required to have >1 year of Medicare Parts A + B + D coverage and be without a recorded history of hip fracture. We ascertained any statin use in the previous year and defined adherent statin use as those who had filled prescriptions for statins to cover >80% of days in that year (proportion of days covered, PDC). We ascertained several potential confounders (demographics, comorbidities, BMI, transplant-related factors) and applied conditional logistic regression with multiple imputation for missing data to estimate odds ratios (OR) and 95% confidence intervals (CI).

**Results:**

We identified 231 hip fracture cases (mean age 51.8 years; 53% female; 11.3% black; 6.9 years from transplant, and 9.9 years from ESRD) and 15,575 matched controls. Any prior statin use was present in 64.1% of cases and 60.3% of controls with 37.2% of cases and 33.9% of controls being found adherent. Unadjusted conditional logistic regression showed an OR of 1.17 (0.89-1.54) for any statin use, and a fully-adjusted OR of 0.89 (0.67-1.19). Compared with statin non-users, the adjusted OR for patients with lesser adherence (PDC ≤80%) and those with greater adherence (PDC >80%) were 0.93 (0.66-1.31) and 0.87 (0.63-1.20), respectively.

**Conclusion:**

Statin use was not associated with hip fracture risk in first-time kidney transplant recipients.

**Electronic supplementary material:**

The online version of this article (doi:10.1186/s12882-017-0559-9) contains supplementary material, which is available to authorized users.

## Background

Hip fractures are devastating events, conferring morbidity, mortality, disability, and high costs to the health care system. In 2005, the general population’s age-adjusted incidence rates were 369 and 794 per 100,000 person-years in men and women, respectively [1]. When compared with the general population, patients on dialysis have been shown to carry a more than 4-fold risk of hip fracture [2]. However, kidney transplant recipients (KTRs) represent a select group of patients that have also maintained a highly elevated hip fracture risk, carrying an even 1.34-fold higher risk than patients on dialysis [3]. The mineral bone disease associated with chronic kidney disease prior to transplant, immunosuppressive regimens utilizing corticosteroids, and age-related osteoporosis are mechanisms that interact to distort bone health. The risk for hip fracture is greatest in the first years post-transplant, declining to equal that of patients on dialysis by the end of the second post-transplant year [3]. Given the increased mortality, significant morbidity, and large healthcare costs associated with hip fracture, any potentially modifiable risk factors including medications should be carefully examined.

Basic and translational studies have identified multiple potential mechanisms by which the 2-hydroxy-3-methylglutaryl coenzyme A reductase inhibitors (statins) affect bone metabolism [4, 5]. Furthermore, statins have been associated with a reduced risk of hip fracture in the 1) elderly general, 2) mostly male Veterans Affairs, and 3) post-menopausal female populations [6–8].

Considering the elevated risk of cardiovascular disease associated with kidney disease and concurrent immunosuppressive medications, statins are widely prescribed for KTRs. Indeed, current guidelines recommend statin treatment for all adult KTRs (leaving some room for discretion in patients aged less than 30 years with fewer cardiovascular risk factors) [9]. A large, retrospective, multicenter international study of kidney transplant outcomes, the Patient Outcomes in Renal Transplantation study, and a separate, prospective observational study at seven transplant centers in the US and Canada estimated that ~40% of patients in the 6 months post-transplant were prescribed a statin [10, 11]. While statins are utilized to diminish this cardiovascular risk in KTRs, the sum of the evidence supporting statin use is not fully conclusive [12]. Therefore, the possibility of added benefit from bone fracture prevention would further justify statin exposure in KTRs. The incidence of hip fractures in first time KTRs has decreased from 1997 to 2010 possibly due to a number of plausible explanations, including adoption of steroid sparing or minimizing protocols, transition to tacrolimus, bisphosphonate use, and cinacalcet use [13]. During the same time, statin use in KTR has also likely increased and may have contributed towards this positive trend.

To our knowledge, there is no data to support an association between statin use and hip fracture risk in the kidney transplant population. We, therefore, conducted the present study to challenge the null hypothesis of no association between statin use and post-transplant hip fracture in U.S. KTRs.

## Methods

### Study design and source population

We conducted a retrospective, nested, matched case control investigation of all first-time KTRs recorded in the United States Renal Data System (USRDS). The USRDS is a national registry that contains detailed information about KTRs via the United Network of Organ Sharing (UNOS) and Medicare claims [14]. First time KTRs who maintained a functioning transplant at any point in time between January 1, 2007 to December 31, 2011 and who had Medicare as their primary health care payer were considered eligible for study. Since diagnoses and prescription information came from Medicare claims, all cases and controls were required to have Medicare Parts A, B, D and be registered for the low-income subsidy for at least 1 year prior to index date.

The date of hip fracture diagnosis was defined as the index date. We identified hip fracture events using the inpatient International Classification of Diseases, 9th Revision (ICD-9) diagnosis codes 820.xx or 821.xx. Additionally, hip fracture cases required any ICD-9 surgical procedure code among 78.55, 79.15, 79.35, 81.52, 81.51, 79.05, 79.25, or 81.40 within 7 days of hip fracture diagnosis [13]. For each case, all available and eligible controls were matched at the index date on transplant vintage (year of transplant), age (within 3 years), sex, and race (non-African American vs. African American). We employed this conditional risk-set matching scheme to maintain parsimony in our outcomes models while controlling for the potential confounding of these previously established risk factors. Parsimony was deemed important to avoid model over-fitting as we had to expect that the number of cases would be small relatively to the number of variables to be considered. Patients with any prior organ transplant or previous evidence of hip fracture were excluded from the study, and controls could subsequently become cases (see Additional file 1: Figure S1).

### Exposure of interest

We focused on statin use as the differentiating exposure. Medicare Part D prescription claims were examined to identify use of any statin and patients’ degree of adherence to these medications. Prior to the index date, any use was defined as at least 1 prescription claim within the last 365 days. Among any statin users, we labeled lesser use among those KTRs who received pharmacy-dispensed pills covering less than 80% of the 365 days prior to the index date. Lastly, higher use was noted as having statin pills dispensed that covered 80% or more of the year prior to the index date.

### Covariates

Patient characteristics were abstracted from the patient and transplant files in the USRDS and comorbidities were ascertained from Medicare claims preceding the index date by ≥1 year (see Additional file 1: Table S1 for specifications). Comorbidities such as diabetes mellitus, cardiovascular disease, cerebrovascular disease, arrhythmia, and rheumatologic disease were considered variables that could potentially contribute to fracture risk either through direct bone modeling or altering patients’ propensity to fall [15–17]. Additionally, age, sex, race, body mass index (BMI), duration of dialysis prior to transplant, type of transplant (living vs. deceased), panel reactive antibody titers, and episodes of rejection were recorded as patient characteristics at time of transplant that could potentially serve as confounders. Medication use, namely types of chronic immunosuppression (mycophenolate mofetil, mycophenolic acid, tacrolimus, cyclosporine, azathioprine, sirolimus or everolimus, and corticosteroids), was identified by use in the year prior to index as found in Medicare Part B and D data.

### Statistical analysis

We used unadjusted and multivariable-adjusted, conditional logistic regression to examine the association of case (hip fracture) vs. control status and prior statin use in first time KTRs. The estimate of association was expressed as odds ratios (OR) and corresponding 95% confidence intervals (CI). Odds ratios were calculated for any vs. non-use as well as for lesser or higher use, respectively, vs. non-use. All of the baseline characteristics and comorbidities detailed in Table 1 (except the factors used for the matching of controls) were included in the multivariate analysis.

**Table 1:** Characteristics of hip fracture cases and matched controls

Variable	Cases (*n* = 231)		Controls (*n* = 15,575)		*P*-value^$^
	Mean or n	±SD or %	Mean or n	±SD or %	
Matched					
Age (years) (±3 years)	51.8	±12.9	51.2	±10.4	0.09
Median (IQR)	54 (42–61)		53 (44–58)		
Male	108	46.7	9308	59.8	-
African American	26	11.3	1126	7.2	-
Time since transplant (±1 year)	6.9	±5.3	4.6	±4.0	0.70
Median (IQR)	6.1 (2.6–10.0)		3.4 (1.3–7.2)		
Not Matched					
Hispanic ethnicity	42	19.0	5143	33.4	<0.01
Missing	10	4.3	184	1.2	
Body mass index (kg/m^2^)	26.0	±5.0	27.3	±5.2	0.01
Median (IQR)	25.0 (22.2–29.3)		26.8 (23.5–30.7)		
Missing	31	13.4	1417	9.1	
Time since ESRD (years)	9.9	±5.2	8.1	±4.4	0.12
Median (IQR)	9.3 (5.7–12.9)		7.4 (4.8–10.6)		
Comorbidities, recorded history of					
Diabetes mellitus	195	84.4	11,594	74.4	<0.01
Cardiovascular disease	204	88.3	11,694	75.1	<0.01
Cerebrovascular disease	101	43.7	3988	25.6	<0.01
Arrhythmia	82	35.5	4205	27.0	0.14
Rheumatologic disease	30	13.0	1382	8.9	0.32
Transplant-related					
Living (vs. deceased) donor	56	24.2	3824	24.6	0.79
Acute rejection, history of	32	13.9	1871	12.0	0.72
Missing	0	0.0	22	0.1	
PRA > 80%	14	7.9	824	6.2	0.59
Missing	53	22.9	2285	14.7	
Immunosuppressant drugs					
Tacrolimus	125	54.1	9807	63.0	0.64
Cyclosporine	71	30.7	2861	18.4	<0.01
MMF/mycophenolic acid	153	66.2	11,138	71.5	0.20
Azathioprine	29	12.6	551	3.5	<0.01
mTor inhibitor	37	16.0	1536	9.9	<0.01
Corticosteroid	187	81.0	10,681	68.6	<0.01
Bisphosphonate use	61	26.4	2111	13.6	<0.01

*ESRD* end-stage renal disease; *MMF* mycophenolate mofetil; *PRA* panel-reactive antibodies

^$^Obtained from a univariate conditional logistic regression model using a complete-case analysis. A *p*-value for male sex and African American race cannot be computed as these variables were hard matched

Since the dataset was expected to contain incomplete data for some variables, we assumed the data to be missing at random and used multiple imputation to create and analyze 40 imputed datasets. [18] Methodologists currently regard multiple imputation as a state-of-the-art technique because it improves accuracy and statistical power relative to other missing data techniques. Incomplete variables were imputed under fully conditional specification and the imputation model included all the variables in the analysis model plus a fixed effect for pair id to account for the matching of controls to cases [19, 20]. Model parameters were estimated applying the analysis model to each imputed data set separately. These estimates and their standard errors were combined using Rubin’s rules.

## Results

### Patient selection and baseline characteristics

We identified 231 cases of hip fractures amongst first time KTRs that fulfilled all stated inclusion and exclusion criteria between 2007 and 2011. We then selected 15,575 control KTRs matched on age, sex, race, and time to transplant. The number of matched KTR controls per case ranged between 1 and 225, with a median of 56 (interquartile range: 29; 93). The mean difference in the matching factors age and time since transplant between cases and controls was 1.72 years and 0.3 years, respectively (Additional file 1: Table S2). These results reflect matching well within the bounds allowed (3 years for age and 1 year for time since transplant). Cases had a higher prevalence of diabetes mellitus, cardiovascular disease, cerebrovascular disease, arrhythmia, and rheumatologic disease. Additionally, cases had higher use of corticosteroids, mTor inhibitors, cyclosporine, and azathioprine (Table 1). In the year prior to index date, 64.1% of cases and 60.3% of controls exhibited any prior statin use. Additionally, 37.2% of cases and 33.9% of controls were denoted as adherent, having had greater than 80% of proportion of days covered (statin prescriptions covering at least 292 out of 365 days preceding the index date) (Table 2).

**Table 2:** Statin use in hip fracture cases and their controls and measures of association

Statin Use Exposure	Cases (*n* = 231)		Controls (*n* = 15,575)		Odds Ratios (95% Confidence Intervals)	
	n	%	n	%	Unadjusted	Adjusted
No Use	83	35.9	6181	39.7	1.0 (referent)	1.0 (referent)
Any Use	148	64.1	9394	60.3	1.17 (0.89–1.54)	0.89 (0.67–1.19)
Lesser Use (PDC <80%)	62	26.8	4111	26.4	1.16 (0.83–1.62)	0.93 (0.66–1.31)
Higher Use (PDC >80%)	86	37.2	5283	33.9	1.18 (0.86–1.61)	0.87 (0.63–1.20)

Note: Separate models were fit to study i) any statin use, or ii) lesser and higher statin use compared with no statin use

From conditional logistic regression models of cases and control sets matched on age (±3 years), sex, race, and time since kidney transplant (±1 year). Adjusted models controlled for Hispanic ethnicity, body mass index, time since incident end-stage renal disease, comorbidities (diabetes, cardiovascular disease, cerebrovascular disease, arrhythmia, rheumatologic disease), transplant related factors (living vs. deceased donor, history of acute rejection, maximum panel-reactive antibody titer), and individual immunosuppressant drugs used in the year prior to the index date (tacrolimus, cyclosporine, mycophenolate mofetil/mycophenolic acid, azathioprine, sirolimus/everolimus, corticosteroids)

The percentage of missing values across the 4 variables incompletely recorded ranged from <0.1% (history of acute rejection) to 14.8% (panel-reactive antibody titer). In total 3549 out of 15,806 records (22%) were incomplete. More cases had PRA missing compared to controls (23% vs. 15%). When performing a complete case analysis of 12,257 patients with all information, an additional 70 case-control groups (representing 2687 patients) were dropped because of no variability in the exposure variable (all statin users or all non-users) within the case-control group. This would leave an analysis sample of only 9570 patients, for a loss of 40% of the originally identified patients, thus further substantiating the use of multiple imputation.

### Associations between statin use and fracture

The unadjusted conditional odds ratio of hip fracture (vs. control) status with any vs. no statin use was 1.17 (0.89–1.54), and those with lesser (<80% of PDC), and higher (>80% PDC) statin use were 1.16 (0.83–1.62), and 1.18 (0.86–1.61), respectively. The multivariable adjusted odds ratios pointed into the opposite direction, but remained non-significant: 0.89 (0.67–1.19), 0.93 (0.66–1.31), and 0.87 (0.63–1.20) for any, lesser, and higher use, respectively (Table 2).

## Discussion

Using a nation-wide registry for KTRs, we found no clear association between the occurrence of a hip fracture event and prior use of statins. We undertook this study based on the findings of experimental research supporting the beneficial effects of statin on bone health and on clinical evidence from observational studies in the general and other sub-populations that supported such putative benefits. We capitalized on the availability of prescription drug information from Medicare Part D, which now permits large-scale studies of medication effectiveness in transplant and other Medicare insured populations. Considering the elevated hip fracture risk in KTRs, any modification of this vulnerability that can be achieved through better understanding of medication effects appears worthwhile.

The mechanisms by which statins can theoretically affect bone health are multifactorial. They have been shown to increase expression of bone morphogenetic protein-2 (BMP-2), resulting in increased osteoblast differentiation and consequent mineralization [21]. Statins may also alter the mevalonate pathway to inhibit protein prenylation and subsequently decrease osteoclast resorption [4]. In conjunction with the known anti-inflammatory effect, these cellular modifications provide a biological plausibility for hip fracture risk attenuation. Several studies support the association between statin use and increased trabecular bone volume [5]. However, the doses utilized in these animal studies, 5 or 10 mg/kg/day of simvastatin, were considerably higher than what is used in clinical practice and may partially explain the inconclusive evidence from observational studies in humans [5].

Previous studies on this topic in the general population have yielded conflicting data. In a study of U.S. Veterans, mostly men, Scranton et al. concluded that patients had a 36% reduced fracture risk if they were prescribed a statin more than once [8]. A population-based, matched case-control study from a large integrated health care organization in Southern California also found a beneficial association between hip fracture status and statin use [adjusted OR 0.68 (0.62–0.74)] [22]. The large-scale Finnish study conducted by Helin-Salmivaara et al. also described favorable outcomes for statin users amongst post-menopausal women age 50–80 [7]. Those patients that reported having used statins for at least 5 years had reduced hip fractures rates compared to controls adherent to hypertension drugs [HR 0.71 (0.58–0.86)] and a randomly selected cohort of the Finnish registry [HR 0.67 (0.55–0.87)]. Of note, there was no association between degree of statin adherence and hip fracture risk. Other observational studies of either cohort or case-control design did not detect any associations. The prospective Women’s Health Initiative study did not identify an association between statin use and fracture risk in 93,716 postmenopausal women [HR 1.22 (0.83–1.81)] [23]. Similarly, van Staa et al. found no association of hip fracture status with prior statin exposure in the large and generalizable United Kingdom General Practice Research Database [OR 0.59 (0.31–1.13)] [24]. Lastly, a Danish national cohort study conducted by Hansen et al. determined an elevated risk of first time fracture within a kidney transplant cohort versus the general background population in multivariate analysis [HR 1.82 (1.62–2.06)]. Lipid-modifying drugs were not associated with fracture risk in the kidney transplant sub-cohort [HR 1.16 (0.87–1.56)]. However, of the 265 observed fractures among KTR only 10% (~27) were hip fractures [25]. Distinguishing type of fracture may be relevant since a recent study found a protective association between statin use and hip fracture, but not with the outcomes of all fractures, lower-extremity fractures, or upper-extremity fractures [26].

While ample information on the efficacy of statins on reducing cardiovascular risk is available, there is limited information from randomized trials regarding their putative effect on fracture rates. Post-hoc analysis of earlier randomized cardiovascular trials had demonstrated a lack of reduced fracture risk [27], but the Justification for the Use of statins in Prevention: an Intervention Trial Evaluating Rosuvastatin (JUPITER) trial was the first a priori examination of this hypothesis. This double-blind placebo-controlled study produced no evidence on the efficacy of high-dose rosuvastatin use for the reduction of fracture risk [HR 1.06 (0.88–1.28)] [28]. However, this trial focused on a rather healthy population that was at much lower fracture risk compared to patients with advanced kidney disease including those with a functioning kidney transplant.

Indeed, bone pathology in kidney disease is quite different from the bone disease experienced by the general population. Uremia, metabolic acidosis, an abnormal vitamin D-parathyroid hormone-FGF-23 axis, and an inflammatory milieu are all factors that contribute to a significantly higher risk of fracture in those with kidney disease. While mitigated after transplant, where some of the physiological processes controlling bone metabolism and health may be reinstated, the effect of prior kidney disease and the long-term use of corticosteroids and other immunosuppressive medications produce a distinct bone disease and render these patients at long-term elevated fracture risk. Thus, for bone disease, evidence garnered from the general population may not be applicable to the kidney transplant population.

There are important differences between the interactions observed with cyclosporine versus tacrolimus and the statins. Co-administration of statin with cyclosporine results in a 6–15 fold increase in statin plasma levels while a number of pharmacological studies have demonstrated relatively little effect of tacrolimus co-administration on statin levels [29–31]. Traditionally, the CNI-statin interaction has been thought to center on competitive inhibition of the CYP3A4 enzyme. However, the important differences observed with cyclosporine versus tacrolimus co-administration on statin plasma levels may be a result of their differential effects on other enzymes important for statin transport and metabolism such as OATP1B1 [32]. In any case, an abundance of caution likely exists around calcineurin inhibitor and statin co-administration. Many of the more potent statins that are also CYP3A4 inhibitors are less frequently employed. Consequently, the alternatively metabolized options, pravastatin and fluvastatin, which are less potent HMG-CoA reductase inhibitors are more commonly prescribed in KTRs. Rosuvastatin, a much more potent non-CYP3A4 inhibiting statin, may be less commonly prescribed amongst KTRs given concerns regarding the development of proteinuria and its higher price [33]. Thus, statins may be relatively ‘underdosed’ in tacrolimus co-treated patients. Unfortunately, our study is to small to support meaningful tests for effect modification or differences in association within class.

In the general population, bone mineral density (BMD) and the fracture risk assessment tool (FRAX) serve as additional calculations to further stratify risk. Due to the aforementioned complex and distinct bone disease in KTRs, the accuracy of these tools is uncertain. The pathology associated with kidney disease is not limited to generalized density and observational studies regarding BMD assessed by dual energy x-ray absorptiometry produce conflicting results [34, 35]. FRAX scores have not been universally accepted for kidney transplants, but an isolated study by Naylor et al. reported predictions for 10-year risk based on FRAX comparable to observed risk [36]. As the investigators noted, a cohort study of 458 patients over a mean of 6 years is not definitive and requires independent validation with larger cohorts. In our study, limited look back windows for the ascertainment of fractures in the more distant past limit the ability to incorporate FRAX into our analysis.

While accounted for in multivariate analysis, cardiovascular disease, diabetes mellitus, cerebrovascular disease, arrhythmias, rheumatologic disease, and steroid use were all more prevalent in cases. Indeed, the direction of the associations swung the opposite way (albeit all without reaching significance) after adjustment for these characteristics, which may motivate speculation that accounting for any residual, unobserved confounding may further push the associations towards statin protection. Clearly, despite being the largest study on the topic to date, at least to our knowledge, similar analyses in larger individual or pooled databases are warranted to achieve further precision on the association of interest.

Other limitations of our study relate to the relatively limited look back window for statin exposure (and for the covariates on immunosuppressant use). This is owing to the relatively recent introduction of prescription drug coverage in Medicare insured patients via Part D. While we could conduct the same study with the requirement of longer prescription drug coverage prior to the index date (e.g., 2 or 3 years), and have attempted to do so, sample sizes would diminish considerably and insufficient numbers of cases would be identified to support adequate multivariable adjustment, thus inducing less precision and increased likelihood of bias. Therefore, we acknowledge not having captured the full duration of statin exposure and being limited to 1 year of statin use ascertainment. Given the possibility that the mechanism of bone protection may require longer periods of statin exposure, our study could have potentially missed a protective effect. Finally, this study was conducted in U.S. KTR with Medicare coverage; its findings may not generalize to KTR with other insurance coverage or in other countries or health care systems.

## Conclusions

In conclusion, this large population-based study of first-time KTRs did not identify an independent association of hip fracture events with any or adherent statin use. These findings need to be interpreted in light of the observational nature of the study as well as the relatively limited number of hip fractures identified, which renders limited power to identify smaller, but clinically meaningful associations.

## Additional file

Additional file 1: Table S1. Code-algorithms Used to Identify Outcomes and Covariables. **Table S2.** Characteristic Differences between Hip Fracture Cases and Matched Controls. **Figure S1.** Flow Diagram of Case Identification and Risk-set Matching. **Table S1.** Provides International Classification of Diseases (Ninth Revision) and Current Procedural Terminology codes utilized to identify comorbidities and outcomes. Table S2 reports the differences in characteristics (demographics, comorbidities, transplant-related features, immunosuppressive drug use) present between hip fracture cases and matched controls within the overall case-control group. **Figure S1.** Diagrams how the 231 hip fracture cases were identified and how risk-set matching determined 15,575 matched controls. (DOCX 101 kb)

## Abbreviations

CNI: Calcineurin inhibitor
ESRD: End-stage renal disease
KTR: Kidney transplant recipients
PDC: Proportion of days covered
USRDS: United States Renal Data System
