# Supplementary material for: Statin use and hip fractures in U.S. kidney transplant recipients
Source: BMC Nephrol. 2017 May 1;18:145. doi: 10.1186/s12882-017-0559-9 (PMC5412039; doi:10.1186/s12882-017-0559-9)

**Supplemental Table S1: Code-algorithms Used to Identify Outcomes and Covariables**

| Descriptions | ICD-9 or CPT Codes |
| --- | --- |
| Fracture neck of femur | 820.XX |
| Fracture of other and unspecified parts of femur | 821.XX |
| Internal fixation (without reduction, closed reduction, open reduction) | 78.55,79.15,79.35 |
| Partial hip replacement | 81.52 |
| Total hip replacement | 81.51 |
| Reduction without internal fixation (closed, open); repair of hip not otherwise specified | 79.05,79.25,81.40 |
| Diabetes mellitus | 249,249.0,249.00,249.01,249.1,249.10,249.11,249.2,249.20,249.21,249.3,249.30,249.31,249.4,249.40,249.41,249.5,249.50,249.51,249.6,249.60,249.61,249.7,249.70,249.71,249.8,249.80,249.81,249.9,249.90,249.91,250,250.0,250.00,250.01,250.02,250.03,250.1,250.10,250.11,250.12,250.13,250.2,250.20,250.21,250.22,250.23,250.3,250.30,250.31,250.32,250.33,250.4,250.40,250.41,250.42,250.43,250.5,250.50,250.51,250.52,250.53,250.6,250.60,250.61,250.62,250.63,250.7,250.70,250.71,250.72,250.73,250.8,250.80,250.81,250.82,250.83,250.9,250.90,250.91,250.92,250.93,357.2,362.0,362.01,362.02,362.03,362.04,362.05,362.06,362.07 |
| Cardiovascular disease, | 414.0,414.00,414.01,414.2,414.3,414.8,414.9,398.91,402.01,402.11,402.91,404.01,404.03,404.11,404.13,404.91,404.93,428,428.0,428.1,428.2,428.20,428.21,428.22,428.23,428.3,428.30,428.31,428.32,428.33,428.4,428.40,428.41,428.42,428.43,428.9,410,410.0,410.00,410.01,410.02,410.1,410.10,410.11,410.12,410.2,410.20,410.21,410.22,410.3,410.30,410.31,410.32,410.4,410.40,410.41,410.42,410.5,410.50,410.51,410.52,410.6,410.60,410.61,410.62,410.7,410.70,410.71,410.72,410.8,410.80,410.81,410.82,410.9,410.90,410.91,410.92,36.1,36.10,36.11,36.12,36.13,36.14,36.15,36.16,36.17,36.19,36.2,414.02,414.03,414.04,414.05,V45.81,00.66,36.06,36.07,36.09,36.0,36.00,36.01,36.02,36.05,V45.82,38.03,38.04,38.05,38.06,38.08,38.33,38.34,38.35,38.36,38.38,38.43,38.44,38.45,38.46,38.48,39.22,39.23,39.24,39.25,39.26,39.29,39.71,440.2,440.20,440.21,440.22,440.23,440.24,440.29,440.3,440.30,440.31,440.32,440.4,440.8,440.9,441,441.0,441.00,441.01,441.02,441.03,441.1,441.2,441.3,441.4,441.5,441.6,441.7,441.9,443,443.0,443.1,443.2,443.21,443.22,443.23,443.24,443.29,443.8,443.81,443.82,443.89,443.9,445,445.0,445.01,445.02,445.8,445.81,445.89,447.1,557.1,557.9,V43.4,84.1,84.10,84.11,84.12,84.13,84.14,84.15,84.16,84.17,84.18,84.19,84.3,411,411.0,411.1,411.8,411.81,411.89,413,413.0,413.1,413.9 |
| Cerebrovascular disease, | 433,433.0433.00,433.1,433.10,433.2,433.20,433.3,433.30,433.8,433.80,433.9,433.90,434,434.0,434.00,434.1,434.10,434.9,434.90,437,437.0,437.1,437.2,437.3,437.4,437.5,437.6,437.7,437.8,437.9,438,438.0,438.1,438.10,438.11,438.12,438.19,438.2,438.20,438.21,438.22,438.3,438.30,438.31,438.32,438.4,438.40,438.41,438.42,438.5,438.50,438.51,438.52,438.53,438.6,438.7,438.8,438.81,438.82,438.83,438.84,438.85,438.89,438.9,430,431,432,432.0,432.1,432.9,433.01,433.11,433.21,433.31,433.81,433.91,434.01,434.11,434.91,436,435,435.0,435.1,435.2,435.8,435.9 |
| Arrhythmia | 427.3,427.31,427.32,426.0,426.12,426.13,426.7,426.8,426.81,426.82,426.89,427.0,427.2,427.81,427.9,37.70,37.71,37.72,37.73,37.74,37.75,37.76,37.77,37.78,37.80,37.81,37.82,37.83,37.85,37.86,37.87,37.89,996.01,V45.01,V53.31,427.1,427.4,427.41,427.42 |
| Rheumatological disease | 710,710.0,710.1,710.2,710.3,710.4,710.5,710.8,710.9,714,714.0,714.1,714.2,714.3,714.30,714.31,714.32,714.33,714.4,714.8,714.81,714.89,714.9,720,720.0,720.1,720.2,720.8,720.81,720.89,720.9,725 |
|  |  |

**Supplemental Table S2: Characteristic Differences between Hip Fracture Cases and Matched Controls**

| Variable |  | mean difference^$^ | | | |  | average proportion of discrepant cases^$^ | | |  |
| --- | --- | --- | --- | --- | --- | --- | --- | --- | --- | --- |
| **Matched** |  |  | | | |  |  | | |  |
| Age (years) (±3 years) |  | 1.7 | | | |  | - | | |  |
| Male |  | - | | | |  | 0 | | |  |
| African American |  | - | | | |  | 0 | | |  |
| Time since transplant (±1 year) |  | 0.3 | | | |  | - | | |  |
|  |  |  | | | |  |  | | |  |
| **Not Matched** |  |  | | | |  |  | | |  |
| Hispanic ethnicity |  | - | | | |  | 0.36 | | |  |
| Missing |  |  | | | |  |  | | |  |
| Body mass index (kg/m^2^) |  | 5.6 | | | |  | - | | |  |
| Missing |  |  | | | |  |  | | |  |
| Time since ESRD (years) |  | 2.7 | | | |  | - | | |  |
|  | | |  |  | | | |  |  | |
| **Comorbidities, recorded history of** | | |  | |  | |  | | |  |
| Diabetes mellitus | | | | - |  | | 0.32 | | |  |
| Cardiovascular disease | | | | - |  | | 0.27 | | |  |
| Cerebrovascular disease | | | | - |  | | 0.46 | | |  |
| Arrhythmia | | | | - |  | | 0.42 | | |  |
| Rheumatologic disease | | | | - |  | | 0.21 | | |  |
| **Transplant-related** | | | |  |  | |  | | |  |
|  | | | |  |  | |  | | |  |
| Living (*vs.* deceased) donor | | | | - |  | | 0.35 | | |  |
| Acute rejection, history of | | | | - |  | | 0.23 | | |  |
| Missing | | | |  |  | |  | | |  |
| PRA > 80% | | | | - |  | | 0.14 | | |  |
| Missing | | | |  |  | |  | | |  |
|  | | | |  |  | |  | | |  |
| **Immunosuppressant drugs** | | | |  |  | |  | | |  |
| Tacrolimus | | | | - |  | | 0.38 | | |  |
| Cyclosporine | | | | - |  | | 0.36 | | |  |
| MMF/mycophenolic acid | | | | - |  | | 0.39 | | |  |
| Azathioprine | | | | - |  | | 0.15 | | |  |
| mTor inhibitor | | | | - |  | | 0.21 | | |  |
| Corticosteroid | | | | - |  | | 0.38 | | |  |
|  | | | |  |  | |  | | |  |
| Bisphosphonate use | | | | - |  | | 0.33 | | |  |

^$^ Between case and controls within case-control group.

Example for correct interpretation of these data: Controls age differed, on average, 1.7 years from that of the corresponding case and an average of 36% of the controls had a Hispanic ethnicity status that was discrepant from that of the corresponding case.

**Supplemental Figure 1: Flow Diagram of Case Identification and Risk-set Matching**


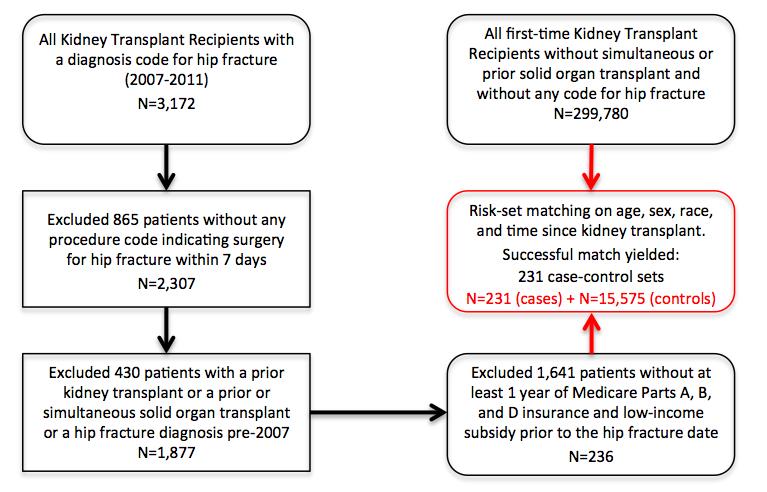

Supplement: Additional file 1: Table S1. — Code-algorithms Used to Identify Outcomes and Covariables. Table S2. Characteristic Differences between Hip Fracture Cases and Matched Controls. Figure S1. Flow Diagram of Case Identification and Risk-set Matching. Table S1. Provides International Classification of Diseases (Ninth Revision) and Current Procedural Terminology codes utilized to identify comorbidities and outcomes. Table S2 reports the differences in characteristics (demographics, comorbidities, transplant-related features, immunosuppressive drug use) present between hip fracture cases and matched controls within the overall case-control group. Figure S1. Diagrams how the 231 hip fracture cases were identified and how risk-set matching determined 15,575 matched controls. (DOCX 101 kb) [file 12882_2017_559_MOESM1_ESM.docx]
